# A 65 nm Duplex Transconductance Path Up-Conversion Mixer for 24 GHz Automotive Short-Range Radar Sensor Applications

**DOI:** 10.3390/s22020594

**Published:** 2022-01-13

**Authors:** Tahesin Samira Delwar, Abrar Siddique, Manas Ranjan Biswal, Prangyadarsini Behera, Yeji Choi, Jee-Youl Ryu

**Affiliations:** 1 Department of Smart Robot Convergence and Application Engineering, Pukyong National University, Busan 48513, Korea; samira.fset@gmail.com (T.S.D.); abrarkhokhar.iiui@gmail.com (A.S.); mrbiswal13@gmail.com (M.R.B.); prangyadarsini.behera@gmail.com (P.B.); wl03845@gmail.com (Y.C.); 2 Department of Electrical and Computer Engineering, University of Waterloo, Waterloo, ON N2L 3G1, Canada

**Keywords:** short-range radar sensor, transmitter, up-conversion mixer, duplex transconductance path, improved cross-quad transconductor

## Abstract

A 24 GHz highly-linear upconversion mixer, based on a duplex transconductance path (DTP), is proposed for automotive short-range radar sensor applications using the 65-nm CMOS process. A mixer with an enhanced transconductance stage consisting of a DTP is presented to improve linearity. The main transconductance path (MTP) of the DTP includes a common source (CS) amplifier, while the secondary transconductance path (STP) of the DTP is implemented as an improved cross-quad transconductor (ICQT). Two inductors with a bypass capacitor are connected at the common nodes of the transconductance stage and switching stage of the mixer, which acts as a resonator and helps to improve the gain and isolation of the designed mixer. According to the measured results, at 24 GHz the proposed mixer shows that the linearity of output 1-dB compression point (OP_1_dB) is 3.9 dBm. And the input 1-dB compression point (IP_1_dB) is 0.9 dBm. Moreover, a maximum conversion gain (CG) of 2.49 dB and a noise figure (NF) of 3.9 dB is achieved in the designed mixer. When the supply voltage is 1.2 V, the power dissipation of the mixer is 3.24 mW. The mixer chip occupies an area of 0.42 mm${}^{2}$.

## 1. Introduction

The rapid growth of communication systems, at millimeter-wavelength (mm-wave) frequencies, has been received considerable attention. In recent years, a particular application area that has attracted a lot of attention is the emerging automotive area at 24 GHz. Since then the frequency bands related to automotive radars have rapidly arisen with enormous market potential as per the declaration of the FCC. Besides radar sensors are considered a vital component of driver assistance systems. It could continually evaluate the environment to help the driver to handle the car efficiently and to prevent collisions [1]. To fulfill this goal, a 24 GHz automotive radar sensor is considered as a leading one, to be employed in smart vehicles. The development of silicon-based IC technologies, allowing the implementation of highly integrated and cost-effective radio designs, has led to recent research activities in the field of automobile radar. Over the last few years, industry and academics have investigated 24-GHz short-range car radar [2,3,4,5,6]. In reality, short-range radar sensors (SRRS) operating at 24-GHz, were previously used in the commercial automobile sector [3]. There is further research and development in the development of long-range 77-GHz and short-range 77–81-GHz silicon radars [7,8,9,10,11]. These articles include K band with highly integrated silicon ICs [12,13,14,15]. The market for mm-wave frequency radios is beginning to evolve and there is great demand for a highly integrated mm-wave transceiver that includes low cost and power. However, the fabrication of mm-wave devices in CMOS processes remains appealing due to high integration and low cost, despite the continued scaling of contemporary CMOS technology [2,3]. The integrated CMOS circuits working at 100 GHz and beyond [4,5,6] are opening up new options.

**Figure 1:**
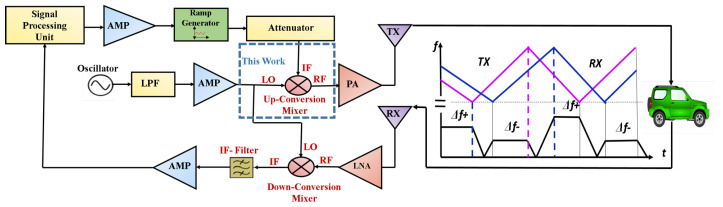
Block diagram of a typical radar transceiver.

Figure 1, represents the block diagram of typical radar transceiver (TRX). The transceiver includes a transmitter (TX), and receiver (RX). In TX the signal from the signal processing unit of SRRS is amplified by an amplifier (AMP), and fed to the ramp generator which passed through the attenuator to generate the intermediate frequency (IF) signal. This IF frequency is mixed in an up-conversion mixer with the local oscillator (LO) signal, to generate the radio frequency (RF) signal. This RF signal is amplified through a power amplifier (PA) and transmitted via a TX antenna. The transmitted signal radiates and reached its target location. The target reflects a portion of the signal back to the TRX. The RX received this reflected signal via the RX antenna. This reflected signal is passed through a low noise amplifier (LNA) to remove the distortions and generate the RF signal. This RF signal is mixed in a down-conversion mixer with the local oscillator (LO) signal, to generate the IF signal. This IF signal is amplified again by AMP and fed back to the signal processing unit of SRRS for further processing.

The mixer is an important block in mm-wave transceiver design to accomplish the frequency transformation. Trades between conversion gain (CG), LO energy, bandwidth, linearity, supply voltage, and power consumption are always required in the design of the mixer. The Gilbert mixer is commonly used in CMOS circuits because of its high CG and minimal LO [4]. In the context of current wireless communication systems, an up-conversion mixer, regarded as an essential circuit block, is used to transform signals from baseband to RF in transmitters. The linearity of a mixer in RF transceivers is crucial and affects the linearity of the system as a whole. In RF systems, nonlinearity creates several difficulties such as gain compression, cross-modulation, intermodulation, etc. To determine the number of gain stages required for a power amplifier, it is crucial to know the linearity characteristic in the design of an up-conversion mixer. Moreover, the input linearity in the up-conversion mixer is usually considerable; thus, a full differential architecture is necessary to remove the LO feed at the output [3]. Besides, 1-dB (P_1_dB) point, help expose a circuit’s potential nonlinearity. 1-dB (P_1_dB) compression point refers to usually operated 1 dB below the compression point to preserve linearity and achieve acceptable efficiency. Therefore, there is a clear trend in wireless applications, to achieve highly-linear, at low power and low voltage. But, the poor linearity of devices at high frequency and inferior noise performance severely restrict the design of mm-wave circuits. Therefore, it is a big challenge to support both the 24-GHz and 38-GHz bands.

### 1.1. Automotive Radar Spectra

**Figure 2:**
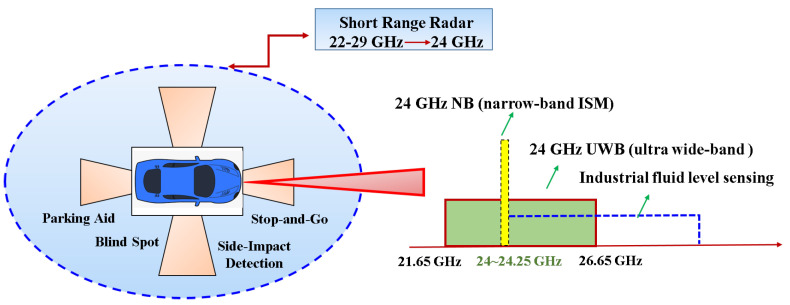
Automobile SRRS applications operate in the 22–29 GHz.

Several frequency bands are assigned for automotive radar applications. Figure 2, depicts automotive radar applications and spectrum allocations that operate in the 22–29 GHz band, including SRRS in particular. The application of 24 GHz SRRS enables features such as blind-spot detection, lane change and parking assistance, and collision avoidance. The 24 GHz band comprises the industrial, scientific, and medical (ISM) band from 24 GHz to 24.25 GHz. In 2002, the FCC indicated that SRRS was permissible within the frequency band of 22 to 29 GHz [1]. With a bandwidth of 250 MHz, this is commonly referred to as narrowband (NB). A 5 GHz ultra-wideband (UWB) is also included in the 24 GHz spectrum. Next, a literature review related to the up-conversion mixer is described.

### 1.2. Literature Review

Many researchers have been taken into consideration to explore the topic of upconversion mixer, within the frequency spectrum of 2.4 GHz to 90 GHz. However, there are few works done in the 24 GHz frequency spectrum, by specifying the particular automobile SRRS applications in the 65-nm CMOS process. Under this literature review segment, in Table 1, we mentioned the all techniques other researchers utilized in their work in the frequency range starting from 24 GHz to 30 GHz, as our work focuses on mainly 24 GHz frequency spectrum.

From the literature review, we could see the existing proposed works has some limitations such as mostly circuits are suffers from poor linearity due to the use of a simple differential amplifier in the input stage. Besides, the other limits such as high current, lacking analytical analysis, and also an essential requirement of a high local oscillator (LO) drive for operation, can considerably increase power consumption and thus affects the overall system. Additionally, in precise, only a few research about 24 GHz CMOS up-conversion mixers have been reported. It is hard to find suitable up-conversion CMOS mixers which can achieve a highly linear, low power, and high conversion gain simultaneously. Mostly, the prior research shows up to now there is no literature available where the improvement of highly-linear and high CG is possible at the same time. Therefore, this research motivates us to apply the proposed techniques named DPT and ICQT to improve the gain and linearity all at once.

Therefore, this research motivates us to apply the proposed techniques named DPT and ICQT to improve the gain and linearity at the same time. To address the highly-linear, A DTP is applied to a mixer in the transconductance stage. The common source (CS) amplifier is used in the main path of DTP, while the enhanced cross-quad transconductor is used in the secondary path (ICQT). At the common nodes of the transconductance stage and switching stage of the mixer, two inductors with bypass capacitors are connected, which operate as a resonator and aid to improve gain and isolation of the designed mixer.

### 1.3. The Main Contributions of the Proposed Up-Conversion Mixer

A 24 GHz automotive short-range radar sensor has been considered by academia and, industry. As a result, next-generation radars may be required to accommodate the 24 GHz band for compatibility and cost savings. Therefore, we have proposed an up-conversion mixer that is best suitable for low-power automotive SRRS applications. Thus, in this section, we have listed the main contributions of our proposed work.

1. In this paper, we propose a highly-linear and high gain 24 GHz up conversion mixer for the automotive radar SRRS applications.
2. It is designed using an enhanced transconductance stage consisting of a duplex transconductance path (DTP) to improve linearity.
3. To achieve the mixer’s high gain and linearity at once, the proposed mixer DTP includes two paths. The first one, MTP holds a common source (CS) amplifier. Besides, the second one, is STP employed as an ICQT.
4. To get a high gain of the proposed mixer besides linearity, we also have applied two inductors with a bypass capacitor in the transconductance and switching stage, which act as a resonator and assist to improve the gain and isolation of the designed mixer.
5. The proposed mixer implemented is to simplify the overall system complexity.

For the simplicity in Table 2, we have described the notations used throughout the work. This paper is divided into four sections. The designed proposed up-conversion mixer is described in Section 2. Section 3 provides measurement results, and Section 4 is the conclusion.

## 2. Proposed Up-Conversion Mixer Design

**Figure 3:**
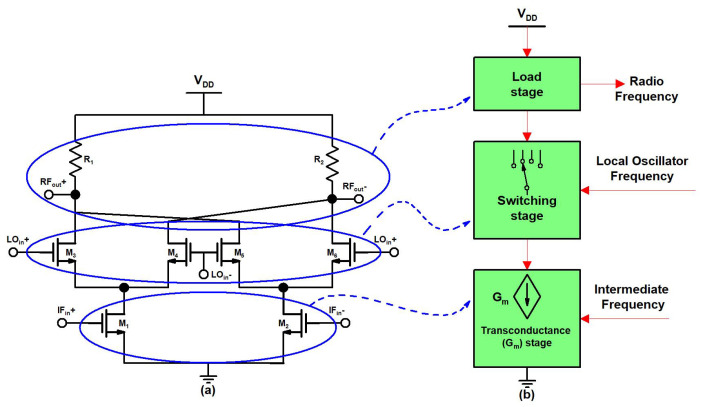
(**a**) Schematic and, (**b**) block diagram of traditional double-balanced Gilbert mixer.

A schematic and block diagram of a traditional double-balanced Gilbert mixer is shown in Figure 3a,b respectively. Due to high CG, a high isolation Gilbert mixer is often used in RF circuits. But, the traditional Gilbert mixer topology suffers from linearity issues, and becomes more worse with low supply voltage and high operational frequency. Furthermore, with high operational frequency, the traditional Gilbert mixer has high noise issues because of parasitic capacitance.

**Figure 4:**
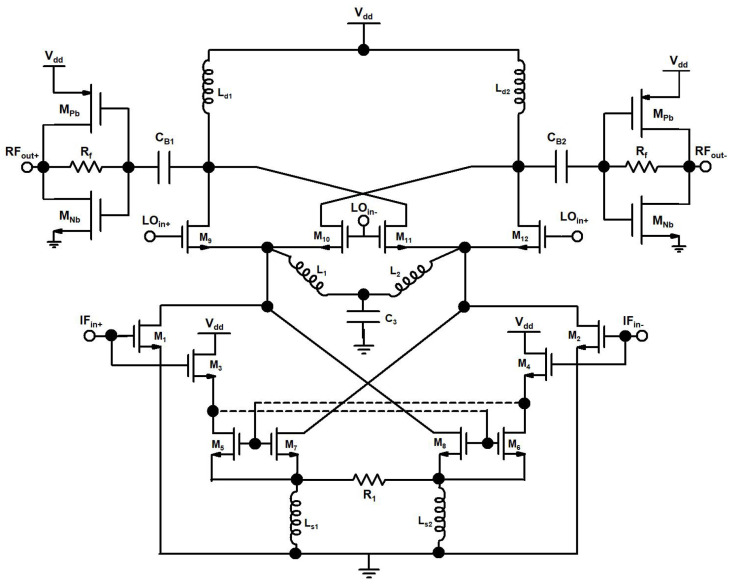
Proposed up-conversion mixer.

A schematic of the proposed up-conversion mixer is shown in Figure 4. The input IF signal is amplified in the DTP stage, consisting of a differential CS amplifier and ICQT. The DTP stage in a designed mixer is implemented to enhance the transconductance and linearity of the mixer. The CS amplifier pair is realized by transistors $M_{1}$, and $M_{2}$. An IF frequency of 2.4 GHz is applied at the gate terminals of $M_{1}$, $M_{2}$ through the input matching network (not shown in the (Figure 4) for simplicity). The ICQT configuration in the designed DTP stage of the mixer consists of transistors $M_{3}$–$M_{8}$ and feedback resistor $R_{1}$ connected between the source terminals of $M_{7}$, and $M_{8}$. In ICQT, the IF is applied at the gate node of transistors $M_{3}$ and $M_{4}$. Transistors $M_{3}$ and $M_{4}$ are cross-coupled with current mirror transistors $M_{5}$–$M_{7}$ and $M_{6}$–$M_{8}$. The resistor $R_{1}$ is connected between the sources of the current mirror and the inductors $L_{s1}$, and $L_{s2}$ act as source degenerated inductors for ICQT. To increase the gain of the designed mixer, inductors $L_{1}$, and $L_{2}$ are implemented with bypass capacitor $C_{3}$ and connected at the common nodes of transconductance and switching stage of the mixer. The amplified and linear output of the DTP stage is connected with the switching stage of the mixer. The switching stage includes transistors $M_{9}$–$M_{12}$ and differential LO input of 21.6 GHz is connected with the gate nodes of switching stage transistors. The switching stage translated the input of the DTP into a 24 GHz differential RF signal. Inductors $L_{d1}$ and $L_{d2}$ act as the load for the RF stage, the push-pull output buffer is designed by complementary n/pMOS transistors $M_{nb}$, $M_{pb}$, and resistor $R_{f}$ for 50 ohm output matching.

**Figure 5:**
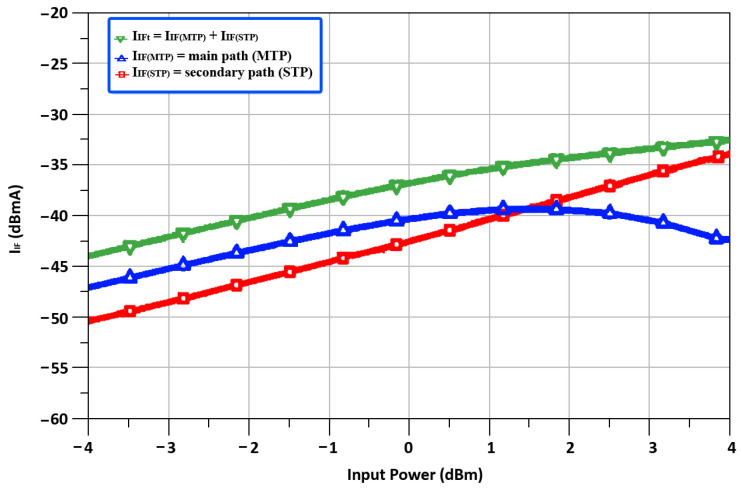
Output currents of duplex transconductance path stage.

**Figure 6:**
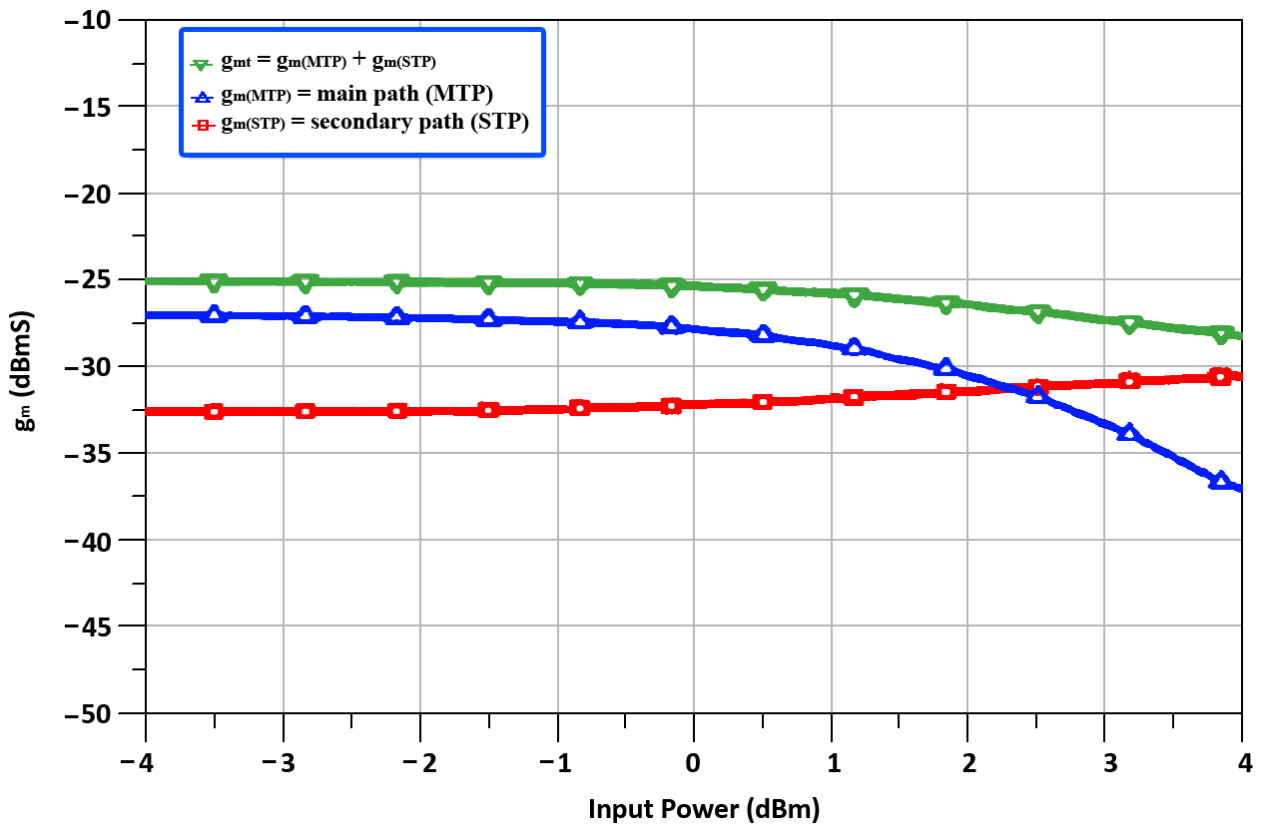
The transconductances of MTP (*g_m_MTP__*) and transconductances of STP (*g_m_STP__*) along with the total transconductances (*g_m_t__*) versus input power.

In the DTP stage of the designed mixer, the two transconductance paths are called the main transconductance path (MTP) and the secondary transconductance path (STP). The MTP is composed of a CS amplifier pair that operates in the saturation region for a high decent gain, while the STP consists of an ICQT. The ICQT-based STP is implemented to boost transconductance and linearity. The ICQT based STP is introduced in the designed mixer because at the high frequency of 24 GHz, linearity is the main concern, and only MTP-based transconductance is not adequate for 24 GHz applications. Figure 4, depicts the output IF currents (*I_IF_MTP__*) of the MTP and the STP (*I_IF_STP__*) together with the total output IF current (*I*_*IFt*_) against the input power, where the input IF frequency is equal to 2.4 GHz. It is seen in Figure 5 that the *I*_*IF*1_ is in saturation, while *I*_*IF*2_ still shows a linear incremental slope as the input signal power level reaches 1.5 dBm. To investigate the characteristics of the designed DTP, the transconductances of MTP (*g*_*m*_*MTP*__) and transconductances of STP (*g*_*m*_*STP*__) along with the total transconductances (*g_m_t__*) contrary to input power are shown in Figure 6. With the rise in the input power, the *g*_*m*_*MTP*__ displays contractive behavior, whereas the *g*_*m*_*STP*__ shows an extensive feature.

**Figure 7:**
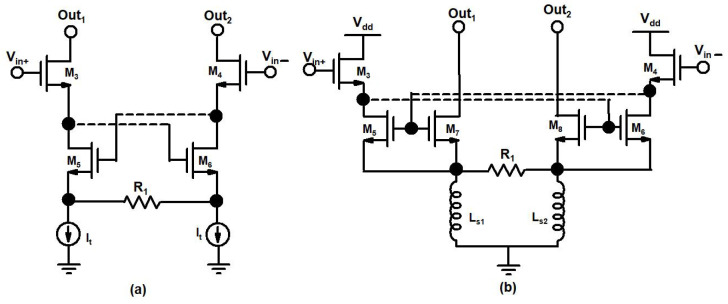
(**a**) Conventional cross-quad transconductor, (**b**) Improved cross-quad transconductor.

In the designed mixer, the secondary transconductance path ICQT of the DTP is used to enhance transconductance and linearity. The conventional cross-quad transconductor is shown in Figure 7a, and the improved cross-quad transconductor is shown in Figure 7b. The conventional cross-quad transconductor (Figure 7a) has been presented to enhance linearity in [31].

**Figure 8:**
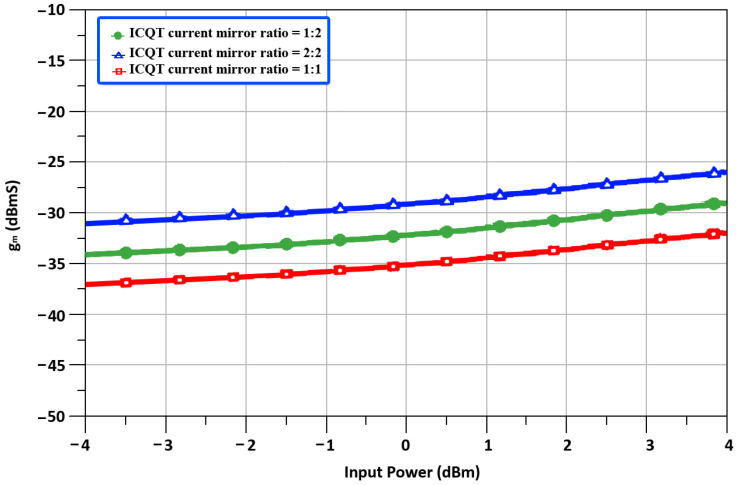
Transconductances of ICQT with different current mirror ratios.

In conventional cross-quad transconductor sources of $M_{5}$, $M_{6}$ exhibits the same voltages and creates a virtual short circuit that is used to shift the burden of linearity away from transistors to a feedback resistor $R_{1}$ and have the transconductor value equal to the value of $\frac{1}{R_{1}}$ and it is dependent on the tail current source. Conventional cross-quad transconductor suffer from positive feedback through parasitic gate-drain (*C_gd_*) capacitance if a load is connected at the drain terminals of $M_{3}$, and $M_{4}$ which may induce instability. Also, in conventional cross-quad transconductors at high frequencies, parasitic inductances lead to negative resistance between the sources of $M_{5}$, $M_{6}$ and the internal resistance of the transistors can also lead to instability. We chose the ICQT for the proposed mixer as a secondary transconductance path to enhance the *g_m_*. In the designed ICQT current mirror transistors $M_{5}$, $M_{7}$, and $M_{6}$–$M_{8}$ are used while positive feedback is avoided and the linearized output signal is taken at the drain terminals $M_{7}$–$M_{8}$. The current mirror transistor ratio ($M_{5,6}$:$M_{7,8}$) is fixed by the ratio of parallel-connected transistors for $M_{5}$ and $M_{7}$ ($M_{6}$ and $M_{8}$). The transconductance of ICQT is independent of tail current sources and it depends on the ratio of current mirror transistors and by setting the mirror ratio to be $M_{7,8}$ > $M_{5,6}$ the ICQT will operate with low power consumption. The transconductances of ICQT with different current mirror ratios are shown in Figure 8.

**Figure 9:**
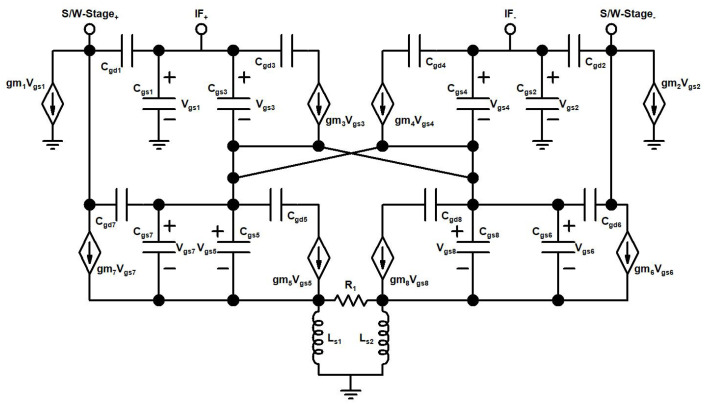
Small signal model of the DTP stage.

A small signal model of the DTP stage of the mixer is presented in Figure 9.

Assuming the input IF voltage signal is equal to *V*_*ifin*+_ = *Acos*ω*_if_in+__t*, the small signal output currents at the drain nodes of transistors $M_{1}$, and $M_{8}$, are shown as follows

(1) $$i_{1}=g_{m1}1V_{ifin+}+g_{m1}2V{{}_{ifin+}}^{2}+g_{m1}3V{{}_{ifin+}}^{3}+...$$

(2) $$\begin{array}{c} i_{8}=\frac{M_{7,8}}{\left(M_{5,6}+M_{7,8}\right)}g_{m8}1V_{ifin+}+\frac{M_{7,8}}{\left(M_{5,6}+M_{7,8}\right)}g_{m8}2V{{}_{ifin+}}^{2}\\ \;\;\;\;\;\;\;\;\;\;\;\;\;\;\;\;\;\;\;\;\;\;\;\;\;\;\;\;\;\;\;\;\;\;\;\;\;\;\;\;\;\;\;\;\;\;\;\;\;\;\;\;\;\;\;\;\;\;\;\;\;\;\;\;\;\;\;\;\;\;\;\;\;\;\;\;\;\;\;\;\;\;\;\;\;+\frac{M_{7,8}}{\left(M_{5,6}+M_{7,8}\right)}g_{m8}2V{{}_{ifin+}}^{3}+... \end{array}$$

(3) $$\begin{array}{c} i_{1}=\frac{g_{m1}2A^{2}}{2}+\left(g_{m1}1A+\frac{3g_{m1}3A^{3}}{4}\right)cos\omega_{ifin+}t+\frac{g_{m1}2A^{2}}{2}cos2\omega_{ifin+}t\\ \;\;\;\;\;\;\;\;\;\;\;\;\;\;\;\;\;\;\;\;\;\;\;\;\;\;\;\;\;\;\;\;\;\;\;\;\;\;\;\;\;\;\;\;\;\;\;\;\;\;\;\;\;\;\;\;\;\;\;\;\;\;\;\;\;\;\;\;\;\;\;\;\;\;\;\;\;\;\;\;\;\;\;\;\;\;\;\;\;\;\;\;\;\;\;\;\;+\frac{g_{m1}3A^{3}}{4}cos3\omega_{ifin+}t+... \end{array}$$

(4) $$\begin{array}{c} i_{8}=\frac{M_{7,8}}{\left(M_{5,6}+M_{7,8}\right)}\frac{g_{m8}2A^{2}}{2}+\frac{M_{7,8}}{\left(M_{5,6}+M_{7,8}\right)}\left(g_{m1}1A+\frac{3g_{m1}3A^{3}}{4}\right)cos\omega_{ifin+}t\\ \;\;\;\;\;\;\;+\frac{M_{7,8}}{\left(M_{5,6}+M_{7,8}\right)}\frac{g_{m1}2A^{2}}{2}cos2\omega_{ifin+}t+\frac{M_{7,8}}{\left(M_{5,6}+M_{7,8}\right)}\frac{g_{m1}3A^{3}}{4}cos3\omega_{ifin+}t+... \end{array}$$

where *g*_*m*1_*n* and *g*_*m*8_*n* are the *n*th transconductances of the transistor $M_{1}$ and $M_{8}$, respectively. In the above analysis, the parasitic effects of CMOS technology are neglected. Hence, the transconductances of the transistors $M_{1}$ (*g*_*m*(*MTP*)_) and $M_{8}$ (*g*_*m*(*STP*)_) can be presented as follows

(5) $$g_{mt\left(MTP\right)}=g_{m1}1+\frac{3g_{m1}3A^{2}}{4}$$

(6) $$g_{mt\left(STP\right)}=\frac{M_{7,8}}{\left(M_{5,6}+M_{7,8}\right)}\left(g_{m8}1+\frac{3g_{m8}3A^{2}}{4}\right)$$

When the transconductance of the MTP and STP add together the total transconductance *g_m_t__* and 1 dB compression point IP_1_dB of the designed mixer, is equal to

(7) $$g_{mt}=g_{m\left(MTP\right)}+g_{m\left(STP\right)}$$

(8) $$g_{mt}=\left(g_{m1}1+\frac{M_{7,8}}{\left(M_{5,6}+M_{7,8}\right)}g_{m8}1\right)+\frac{3A^{2}}{4}\left(g_{m1}3+\frac{M_{7,8}}{\left(M_{5,6}+M_{7,8}\right)}g_{m8}3\right)$$

(9) $$IP_{1dB}=\sqrt{0.145\left|\begin{array}{cc} \; & \frac{g_{m1}1+\frac{M_{7,8}}{\left(M_{5,6+}M_{7,8}\right)}g_{m8}1}{g_{m1}3+\frac{M_{7,8}}{\left(M_{5,6+}M_{7,8}\right)}g_{m8}3}\\ \; & \end{array}\right|}$$

The degeneration resistor $R_{1}$ of the ICQT and mirror ratio is adjusted so that high transconductance is obtained. The maximum linearity is obtained by using the ICQT. To attain linearity, the DTP structure is more power-hungry than the conventional transconductance stage of the mixer, due to current mirror transistors. But with the incremental current of the maximum obtainable linearity in the DTP structure is higher as compared to the conventional mixer.

## 3. Results and Discussion

**Figure 10:**
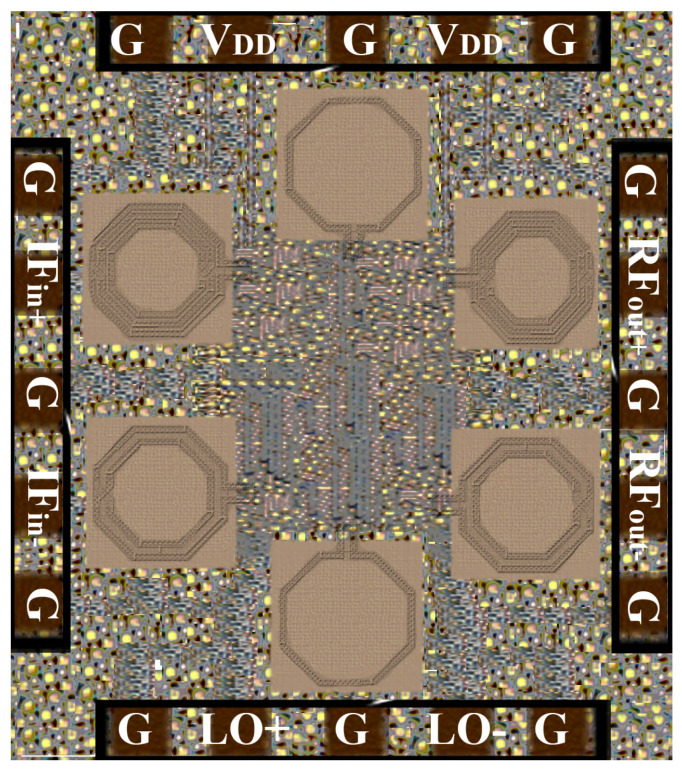
Micro-photograph of designed mixer’s chip.

The designed mixer, based on the DTP scheme, was fabricated with 65 nm CMOS technology. A micro-photograph of the designed mixer with a chip size of 0.42 mm${}^{2}$ (0.72 × 0.59 mm${}^{2}$), is shown in Figure 10. While carefully doing mixer chip layout, all design rules are considered to reduce the inductive, capacitive, and resistive parasitic effects of diffusion strips, and interconnection metal lines, and also to reduce a mixer with performance that does not deviate much from the schematic simulations. All the transistors in the layout are implemented with multi-fingers to reduce the poly layer and gate resistance and also it reduces the non-linear capacitance due to diffusion strips. Multiple metal layers are used to create the low resistive and inductive ground path of the designed mixer. The electromagnetic coupling effects of inductors in the mixer circuit are decreased by implementing a ground layer underneath the coil of an inductor.

The designed mixer’s operational characteristics at 1.2 DC supply voltages were measured with ground-signal-ground-signal-ground (GSGSG) measuring probes and it consumes 3.24 mW power. The signal generator (R&S SMF100A) and vector network analyzer (Keysight Agilent N5247A) were used to feed the input signals at the LO port and IF port, respectively, and a spectrum analyzer (R&S Fsu67) was used to measure the output signal at the RF port.

**Figure 11:**
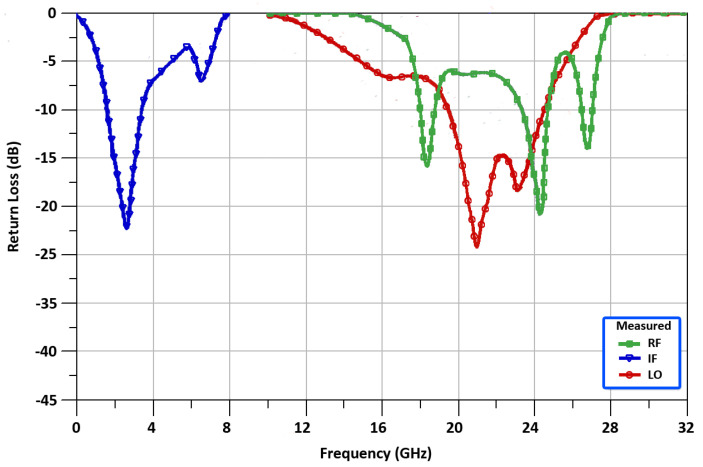
The Mixer’s measured return loss.

Figure 11 shows, the measured return loss of −21.5, −22.45, and −24.7 dB at the RF, IF, and LO ports, respectively. For RF and LO return loss simulation frequency range is 10 GHz to 32 GHz, and for IF return loss simulation frequency range is 0 GHz to 8 GHz.

**Figure 12:**
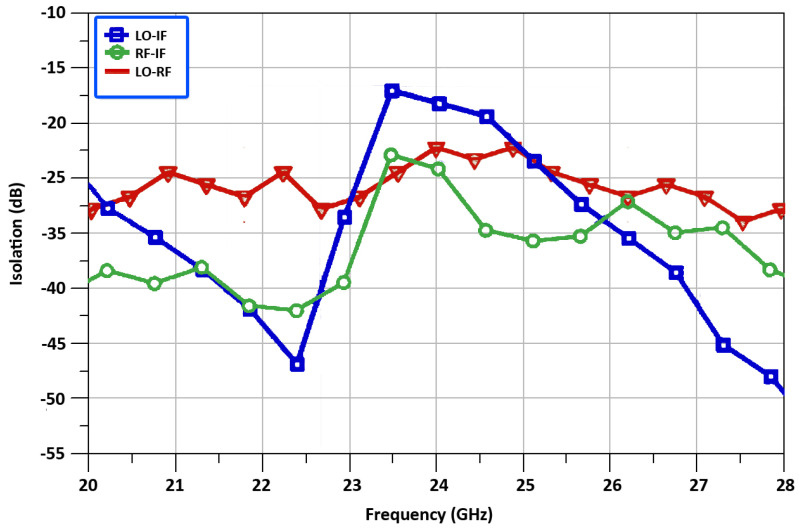
Isolations between the mixer’s ports.

The measured isolation between LO-RF port, RF-IF port, and LO-IF port is equal to −22.4, −24.3, and −18.1 dB, respectively, at 24 GHz and is depicted in Figure 12. All of these results are well matched within the operating frequencies and illustrate the good performance of the designed mixer.

**Figure 13:**
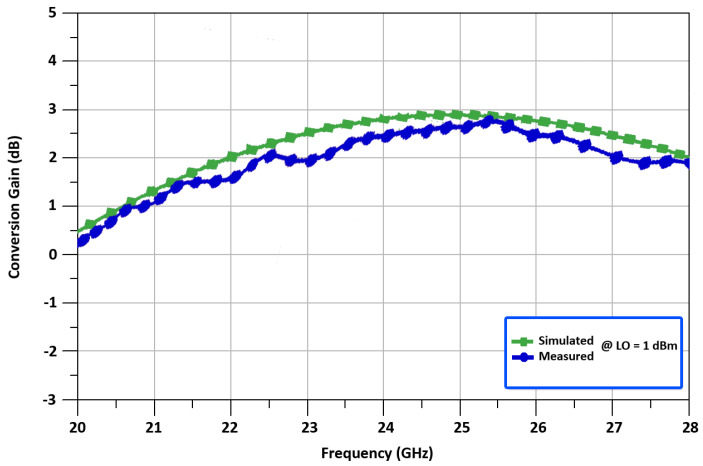
Conversion gain with respect to frequency of the mixer.

**Figure 14:**
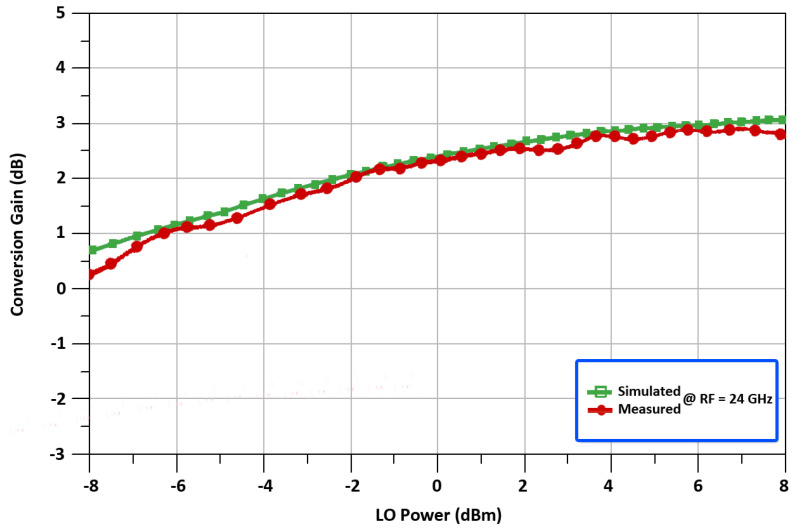
Conversion gain with respect to LO power.

Figure 13 shows the measured result of conversion gain vs. frequency for the 24 GHz up-converted mixer. While the RF output frequency is equal to 24 GHz, the mixer realizes a conversion gain of 2.49 dB in Figure 13. To further analyze the characteristics of the proposed mixer, Figure 14 represents the measured CG vs. LO power for the 24 GHz up-converted mixer. The result shows that the CG improves as the LO power is increased. However, the LO power, on the other hand, has been deliberately chosen to be 1 dBm for low power operation. It demonstrates that when the LO power is more than 0 dBm, the proposed mixer can provide a large CG. In order to achieve a tradeoff between LO power and CG, the necessary value of 1 dBm for LO power is determined, with a CG of 2.49 dB. It’s worth noting that too much LO power can deteriorate the linearity of the designed mixer.

**Figure 15:**
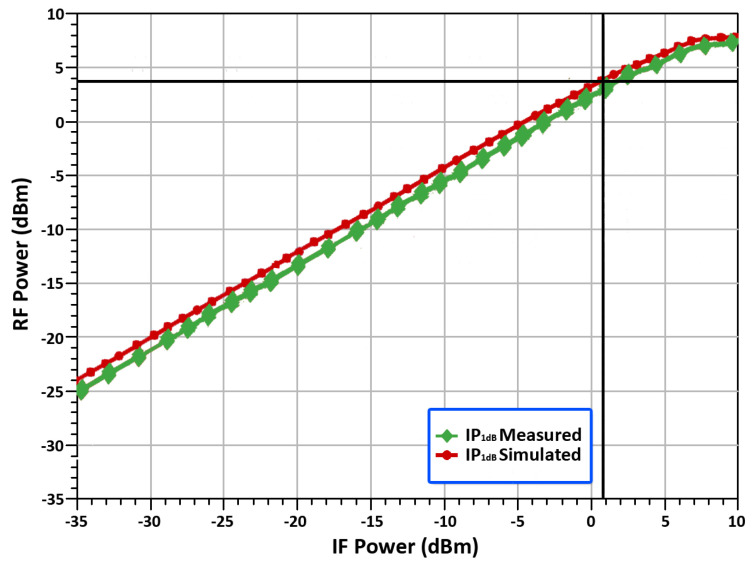
RF output power vs. IF input power.

Additionally, for the efficacy of the proposed mixer, we measured linearity. 1 dB compression point (IP_1_dB) is one of the most important specifications for the designed mixer, as it is considered as an ideal indication to operate the circuit linearly. In this case, the output power level deviates from the ideal power level by 1 dB. Once a designed mixer reaches its IP_1_dB it goes into compression and becomes non-linear, producing distortion, harmonics, and intermodulation products. Thus, the designed mixer should always be operated below the compression point. On that note, we have shown the measured result of the linearity, RF output power versus IF input power in Figure 15. It is clearly seen from Figure 15 that, the designed mixer achieved the IP_1_dB is equal to 0.9 dBm, while the RF frequency is 24 GHz. To understand the nonlinear behavior, the IP_1_dB of the simulated one was 0.9 dBm, the OP_1_dB showed 3.9 dBm respectively. Overall, the mixer showed good linear performance within the 24 GHz frequency range.

**Figure 16:**
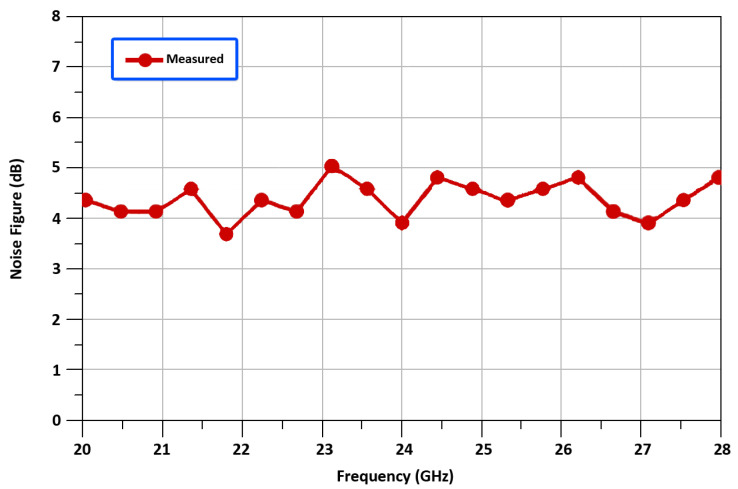
Measured noise fig. vs. RF frequency.

**Figure 17:**
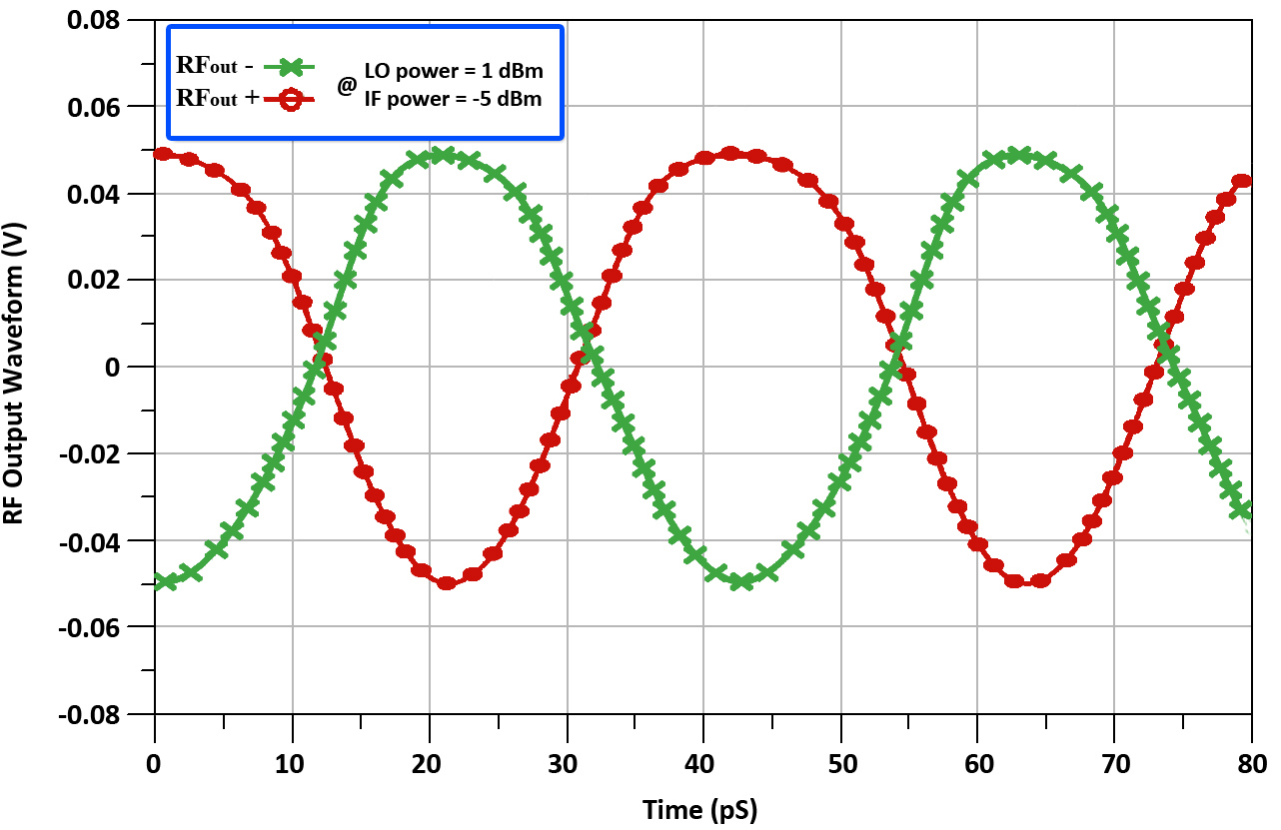
Output voltage waveform.

Figure 16 and Figure 17, shows the measured noise fig. (NF), and the output voltage waveform exhibiting a RF of 24 GHz. In Figure 16, the mixer acquires a minimal NF of 3.9 dB. It can be observed in Figure 17, that the mixer’s RF signal has a peak-to-peak voltage swing of 90 millivolts, when the input LO power and IF power are equal to 1 dBm and −5 dBm respectively. This differential up-converted output signal is achieved at the RFout+ and RFout-terminals of two push/pull output buffers used in the proposed mixer design. These push/pull output buffers help to match 50 Ohm output matching and also aids in the formation of a nearly pure sinusoidal output signal. Table 3, shows the comparison summary for recently reported results.

### Implications and Findings of the Proposed Work

The mixer is one of the most important components of a transmitter system, and its performance has a significant impact on the transmitter’s overall operation. On that note, our implications and findings show that significant improvement while achieving the highly linear and high gain of the proposed up-conversion mixer. These findings suggest that how the dual transconductance path influences the mixer’s most fundamental parameter. In our work, a very significant observation is that there is a good agreement between the trend of simulation and measurement, which verifies the feasibility of improving the conversion gain, linearity of the improved cross-quad transconductor, and duplex transconductance path respectively. Moreover, the operational properties of the proposed mixer were examined with ground-signal-ground-signal-ground (GSGSG) measuring probes at 1.2 DC supply voltages. Also, the mixer consumes 3.24 mW of power.

## 4. Conclusions and Future Research

A 24 GHz up-conversion mixer using 65 nm CMOS technology is proposed for automotive short-range radar sensor applications. This paper aimed to increase mixer linearity in the 24 GHz frequency range. Therefore, we proposed a mixer with an enhanced transconductance stage consisting of a duplex transconductance path (DTP) to improve linearity. The DTP comprises two-path named MTP which includes a common source (CS) amplifier, and the other is STP implemented as an improved cross-quad transconductor (ICQT). Two inductors with a bypass capacitor are connected at the common nodes of the transconductance stage and switching stage of the mixer, which act as a resonator and help to improve the gain and isolation of the designed mixer. The measured IP_1_dB of the designed mixer is 0.9 dBm, with a conversion gain of 2.49 dB at 24 GHz, and a noise fig. of 3.9 dB at 24 GHz. The mixer only consumes 3.24 mW at 1.2 V. We believe that the proposed mixer has highly linear, and low DC power consumption at 24 GHz, and is best suitable for low-power automotive SRRS applications.

In the future, research will be conducted to design a complete 24 GHz CMOS transmitter front-end by using newly proposed mixer topologies. Besides, we can do more work on the design reliability of the mixer in terms of time, budget, and demanding profile constraints. Furthermore, to be specific, automotive radar applications, have pushed for more integration, as well as multi-mode and multi-band operation, to offer low-cost, high-functionality consumer devices. As a result, more research is required to improve the efficiency of multiple-mode phased arrays in the 24 GHz bands, which will eventually allow for combined short-range and long-range detection on a single chip.

## Figures and Tables

**Table 1:** Literature review.

Ref.	Frequency Technology	Methodology	Result	Remarks
Chiou et al. [16]	17.5–22.3 @ 90 nm	Current mirror reused topology	CG: −0.62 dB OP_1_dB: −13 dBm P_DC_ 0.149 mW	Circuit suffers from poor linearity
Won et al. [17]	24 @ 130 nm	Adaptive biasing scheme	CG: −1.9 dB OP_1_dB: 0.3 dBm P_DC_: 22.8 mW	Circuit gives a small IF bandwidth
Chen et al. [18]	27.5–43.5 @ 65 nm	A linearized g_m_ stage using coupled resonators	CG: −5 dB OP_1_dB: 0.4 dBm P_DC_: 14 mW	It shows an OP_1_dB of 0.42 dBm
Siddique et al. [19]	24 @ 65 nm	Neutralization technique	CG: 4.7 dB OP_1_dB of 0.42 dBm P_DC_: 5.2 mW	Lacking analytical discussion.
Siddique et al. [20]	24 @ 65 nm	An I-DS technique	CG: 6.5 dB OP_1_dB of 0.41 dBm P_DC_: 4.9 mW	Focused to achieve highly-linear, while ignore the effects of CG.
Huynh et al. [21]	24 @ 65 nm	Double balanced Gilbert mixer	CG: 6.5 dB IIP3: 15.3 dBm P_DC_: 6.8 mW	Has high current of 40 mA
Lai et al. [22]	20–26 @ 90 nm	Multi-layer marchand balun	CG: 2 dB IIP3: −14.8 dBm P_DC_: 11.1 mW	Achieves a CG of 2 dB @ 22.1 GHz
Verma et al. [23]	22–29 @ 130 nm	Dual-gate mixer	CG: −2 to−0.7 dB OP_1_dB: −7 to 5.2 dBm P_DC_: 8.0 mW	P_DC_ shows almost 8.0 mW
Qayyum et al. [24]	24–32 @ 130 nm	Gilbert mixer transformer baluns	CG: 13.7 @ 26.5 dB OP_1_dB: 1.46 @ 28 P_DC_ 90 mW	High CG, 13 dB @ 28 GHz
Byeon et al. [25]	27.5–43.5 @ 65 nm	Complementary DS technique	CG: 11.4 dB OP_1_dB: 2 dBm P_DC_: 15 mW	Improves the LO leakage and power capability performances
Chen et al. [26]	27.5–43.5 @ 65 nm	Applied TPTS I/p and O/p baluns	CG: −5 dB OP_1_dB: 0.42 dBm P_DC_: 14 mW	Impedance matching and linearity is good
Syu et al. [27]	27.5–43.5 @ 0.18 μm	Utilizing n/pMOS TCAs	CG: −3 dB OP_1_dB: −11 dBm P_DC_ < 6.8 mW	More DC power is required
Comeau et al. [28]	28 @ 0.18 μm	Series connected triplet	CG: −0.8 dB IIP3: 2.2 dBm	Accommodates a larger input power, and enhances the SNR.
Lin et al. [29]	28.1 @ 0.18 μm	Resistive mixer comprises LO boosting linearization technique	CG: −8.5 dB OP_1_dB: −2.7 dBm P_DC_: 0 mW	Zero dc power consumtion
Tsai et al. [30]	15–34 @ 0.18 μm	A weak inversion biasing technique	CG: 3.5 dB OP_1_dB: −21.2 dBm @ 28 GHz P_DC_: 2.472 mW	Demonstrates flat CG and low dc power

**Table 2:** Notations used in the proposed work.

Definition	Notation
Main transconductance path	MTP
Secondary transconductance path	STP
Duplex transconductance path	DTP
Improved cross-quad transconductor	ICQT
Noise fig.	NF
1-dB compression point	IP_1_dB
Output 1-dB compression point	OP_1_dB
Conversion gain	CG
Short-range radar sensor	SRRS
Millimeter-wavelength	mm-wave
Local oscillator	LO
Ultra-wideband	UWB
Narrowband	NF
Common source	CS
IF currents of the MTP	*IIF*_*MTP*_
IF currents of the STP	*IIF*_*STP*_
The total output IF current	*IIF_t_*
Transconductances of MTP	*gm*_*MTP*_
Transconductances of STP	*gm*_*STP*_
Total transconductance	*g_m_t__*

**Table 3:** Comparison summary for recently reported results.

Ref.	[16]	[17]	[20]	[22]	[26]	[29]	[32]	[33]	[34]	This Work
Freq. (GHz)	17.5–22.3	23.4–29.2	24	20–26	27.5–43.5	28	24	60	17	24
Tech. (nm)	90	130	65	90	65	180	180	65	130	65
OP_1_dB	−13	−0.3	4.1	−14.8	0.42	−2.7	NA	−5	4.2	3.9
CG (dB)	−0.62	−1.9	4.1	2	−5	−8.5	13	−6.5	−4	2.49
P_DC_ (mW)	0.149	22.8	4.9	11.1	14	0	4	29	93	3.24
Chip Area (m${}^{2}$)	NA	NA	0.4	0.37	0.686	0.34	NA	0.27	0.5	0.42

## Data Availability

Not applicable.
